# Single Low Dose of Cocaine–Structural Brain Injury Without Metabolic and Behavioral Changes

**DOI:** 10.3389/fnins.2020.589897

**Published:** 2021-01-22

**Authors:** Camilla Nicolucci, Mariana Lapo Pais, A. C. Santos, Fabiana M. Ribeiro, Pedro M. C. C. Encarnação, Ana L. M. Silva, I. F. Castro, Pedro M. M. Correia, João F. C. A. Veloso, Julie Reis, Marina Z. Lopes, Maria F. Botelho, Frederico C. Pereira, Denise G. Priolli

**Affiliations:** ^1^Multidisciplinary Research Laboratory, São Francisco University Post-graduation Stricto Sensu Programme, Bragança Paulista, Brazil; ^2^Faculty of Sciences and Technology, University of Coimbra, Coimbra, Portugal; ^3^Faculty of Medicine, Institute of Biophysics, University of Coimbra, Coimbra, Portugal; ^4^Faculty of Medicine, Coimbra Institute for Clinical and Biomedical Research, University of Coimbra, Coimbra, Portugal; ^5^Center for Innovative Biomedicine and Biotechnology, Coimbra, Portugal; ^6^Department of Physics, Institute for Nanostructures, Nanomodelling and Nanofabrication (i3N), University of Aveiro, Aveiro, Portugal; ^7^Radiation Imaging Technologies Lda, Ílhavo, Portugal; ^8^Multidisciplinary Research Laboratory, São Francisco University Scientific Initiation Programme, Bragança Paulista, Brazil; ^9^Faculty of Medicine, Institute of Pharmacology and Experimental Therapeutics, University of Coimbra, Coimbra, Portugal

**Keywords:** cocaine, brain damage, metabolic imaging, histological change, behavior

## Abstract

Chronic cocaine use has been shown to lead to neurotoxicity in rodents and humans, being associated with high morbidity and mortality rates. However, recreational use, which may lead to addictive behavior, is often neglected. This occurs, in part, due to the belief that exposure to low doses of cocaine comes with no brain damage risk. Cocaine addicts have shown glucose metabolism changes related to dopamine brain activity and reduced volume of striatal gray matter. This work aims to evaluate the morphological brain changes underlying metabolic and locomotor behavioral outcome, in response to a single low dose of cocaine in a pre-clinical study. In this context, a Balb-c mouse model has been chosen, and animals were injected with a single dose of cocaine (0.5 mg/kg). Control animals were injected with saline. A behavioral test, positron emission tomography (PET) imaging, and anatomopathological studies were conducted with this low dose of cocaine, to study functional, metabolic, and morphological brain changes, respectively. Animals exposed to this cocaine dose showed similar open field activity and brain metabolic activity as compared with controls. However, histological analysis showed alterations in the prefrontal cortex and *hippocampus* of mice exposed to cocaine. For the first time, it has been demonstrated that a single low dose of cocaine, which can cause no locomotor behavioral and brain metabolic changes, can induce structural damage. These brain changes must always be considered regardless of the dosage used. It is essential to alert the population even against the consumption of low doses of cocaine.

## Introduction

Drug dependency is considered a physical and psychological condition that induces chronic and recurrent diseases. The continued use of psychoactive substances can cause functional changes in the brain (Dias et al., 2008). Cocaine is one of the most widely used drugs in the world. The European Drug Report of 2020 showed that about 4.3 million people between 15 and 64 years old used cocaine in 2018, and 17.9 million had used it at least once (European Drug Report, 2020). In 2016, the number of young people who have already had any contact with illicit drugs was 236,800 (European Drug Report, 2016). Its consumption reaches about 0.4% of the world population, and most users (70%) are concentrated in the American continent (Gootenberg, 2019).

It is well-established that cocaine is a psychostimulant (Kalivas, 2007). This drug inhibits the reuptake of monoamine neurotransmitters, including dopamine (DA) and noradrenaline (NA). The DA increase occurs in the mesocorticolimbic system (the brain reward system), which is responsible for the well-being sensation and euphoria, thus playing a major role in the addiction process (Planeta et al., 2013). DA auto-oxidation can lead to oxidative stress and apoptosis (Dias et al., 2008; Planeta et al., 2013). There is evidence that oxidative stress contributes to cocaine neurotoxicity (Dietrich et al., 2005; Pereira et al., 2015). Changes in brain circulation triggered by cocaine use led to additional brain injury risk (Niu et al., 2019). Browndyke et al. (2004) demonstrated that these blood flow abnormalities might be related to cognitive impairments reported in cocaine-dependent populations (Browndyke et al., 2004). Moreover, cerebrovascular accidents rank amongst the most severe adverse events from cocaine abuse (Sordo et al., 2014).

Pre-clinical neuroimaging studies aiming to model human diseases and traits have been increasing in the last decade (Volkow et al., 1997; Moreno-López et al., 2012; Caprioli et al., 2013; Hanlon et al., 2013; Cannella et al., 2017; Nicolas et al., 2017). In an animal model, individuals can be followed up longitudinally over time, allowing the study of disease progression, development of compensatory changes, and long-term evaluation of the safety and efficacy of interventions (Zaidi, 2014; Cannella et al., 2017; Nicolas et al., 2017). In particular, pre-clinical positron emission tomography (PET) plays a fundamental role, not only in the validation of animal models of human brain disease but also in the quantitative measurement of regional changes in brain activity. These regional alterations in cerebral sub-regions are affected by diseases or psychoactive agents such as drugs of abuse. There is also evidence that repeated administration of a psychostimulant drug in laboratory animals may cause a change of different parameters, including cerebral glucose metabolism, in opposition to the one caused by an acute administration (Hammer and Cooke, 1994; Zocchi et al., 2001). The selectivity of glucose metabolism changes in the basal ganglia and prefrontal cortex (PFC) suggests that regional metabolic changes, observed in cocaine users during detoxification, are related to changes in the DA activity in the brain (Volkow et al., 1997). Several studies used brain imaging techniques to investigate the changes in brain activity induced by drugs (Hammer et al., 1993; Gould et al., 2009; Caprioli et al., 2013; Hanlon et al., 2013). Particularly, PET studies using 2-deoxy-2-[^18^F]fluoro-d-glucose (^18^F-FDG) have demonstrated abnormal brain glucose metabolism connected to cocaine addiction and withdrawal. The ^18^F-FDG is a widely used radiotracer in PET due to its convenient half-life (110 min) and its well-established role in glycolytic metabolism (Alavi and Reivich, 2002; Caprioli et al., 2013; Hanlon et al., 2013; Cannella et al., 2017; Nicolas et al., 2017). Acute withdrawal in cocaine addicts is associated with a glucose metabolic rate higher than drug-naïve controls or cocaine abusers tested in late withdrawal (Volkow et al., 1991). Other researchers discovered a negative correlation between the severity of cocaine use and the glucose metabolic rate (Moreno-López et al., 2012). Regarding pre-clinical models, some authors evaluated the metabolic activity changes after short (1 week) and long (4 weeks) periods of cocaine abstinence in rats with a history of cocaine self-administration, using the escalation model (Nicolas et al., 2017). They showed that escalation of cocaine self-administration produced cerebral changes that are quantitatively and qualitatively different from those found after short access to cocaine self-administration; i.e., the changes in basal brain metabolic activity depend on the intensity of cocaine self-administration and the duration of abstinence (Nicolas et al., 2017). Although there are a growing number of neuroimaging studies in cocaine addiction settings, there are no neuroimaging studies in the context of a single low dose of cocaine. Additionally, data on the potential for brain injury induced by the consumption of low doses of cocaine remain scarce (Volkow et al., 1997; Heard et al., 2008).

The present work puts forward the hypothesis that even a single low dose of cocaine can cause deleterious brain changes. Therefore, this work aims to evaluate locomotor behavioral, metabolic, and morphological brain data of mice exposed to a single low dose of cocaine.

## Materials and Methods

### Subjects and Housing Conditions

Male Balb-c mice, with a mean age of 6 weeks and an average weight between 20 and 30 g, were used in this study. The Multidisciplinary Research Laboratory of the University of São Francisco, Bragança Paulista (Brazil), in collaboration with both the Biophysics and Pharmacology and Experimental Therapeutics Institutes of the Faculty of Medicine of University of Coimbra (Portugal), developed the research. All experiments were conducted following the European Union directives (86/609/EEC) for the care of laboratory animals and the iCBR *Vivarium* guidelines. The project is under the ORBEA 17/2015 and the DGAV authorization.

### General Experimental Design

**Figure 1:**
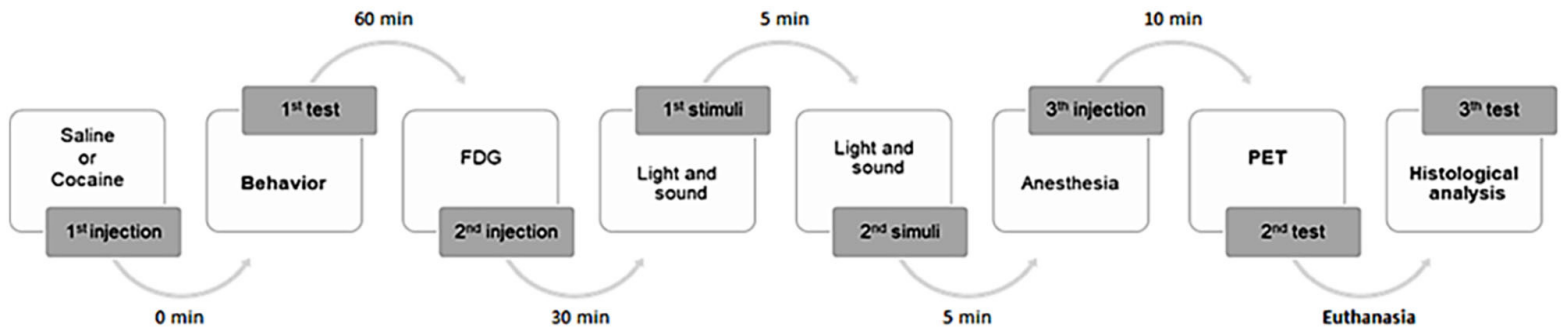
General experimental design. A total of 18 animals were used, randomly divided into two groups: mice *non-exposed* (*n* = 8) or mice *exposed* (*n* = 10) to a single low dose of cocaine. Mice were submitted to a behavioral study. Five animals from each group were subsequently studied by PET imaging analysis. To histological analysis, six randomized mice brain samples were collected.

Our general experimental design is presented in Figure 1. A total of 18 animals were used, being randomly divided into two groups: mice *non-exposed* (*n* = 8) or mice *exposed* (*n* = 10) to a single low dose of cocaine (Merck, Darmstadt, Germany). The *non-exposed* animals represent the controls that were injected intraperitoneally (i.p.) with saline (0.9% NaCl, 0.5 ml). The *exposed group* was injected (also i.p.) with cocaine (0.5 mg/kg, 0.5 ml). The dose was chosen based on the lowest dose having demonstrated dopaminergic visible action on PET imaging evaluation in human volunteers (Heard et al., 2008), although promoting changes in brain neurochemistry (Volkow et al., 1997).

Animals were submitted to a behavioral test followed by PET imaging; and brains were collected. Initially, saline or cocaine was administered to the animals, which subsequently underwent a behavioral test for 60 min. Afterwards, five randomized mice from each group were injected (i.p.) with ^18^F-FDG. The radiotracer had an uptake period of 50 min post-injection. Subsequently, animals were anesthetized (i.p. injection) [0.2 ml of a mixture of ketamine (1.5 mg/mg weight) + chlorpromazine (0.05 mg/mg weight) (3:1):saline (1:1)] 10 min prior to the PET imaging acquisition (+/−30 min). For routine histological technique analysis, anesthesia (ketamine + chlorpromazine) overdose was induced in three randomized mice from each group for brain sample collection.

### Behavioral Study

The open field maze (OFM) test has been used to assess general motor activity and anxiety (Gould et al., 2009; Kraeuter et al., 2019). The animals were allowed a habituation period of 45 min to the behavioral test room before the OFM test procedure. A soundproof test room was used. Moreover, the behavioral test was performed under a white noise (80 dB) stimulus to further attenuate sound interference (Henry et al., 2010). Additionally, the light level inside the OFM was maintained at 7–8 lux. Following saline or cocaine administration, animals were immediately placed in the middle [AS4] of the open field, and motor activity was monitored through a video camera positioned above the apparatus. The images were analyzed later with the ANY Maze video tracking (Stoelting Co., Wood Dale, IL, USA) by a researcher who was unaware to which experimental group the animals belonged to. The animals were allowed to move freely in the OFM for 60 min. The OFM evaluation was performed by analyzing the following parameters: (a) total walked distance; (b) mean speed; (c) maximum speed; (d) periphery distance; (e) time spent in the periphery; (f) latency time to center; g) the number of entrances in the center; (h) center distance; and (i) time spent in the center.

### Positron Emission Tomography Imaging Study

A metabolic PET scan with ^18^F-FDG was performed, under basal conditions in fasted animals (6–8 h), to study the cerebral metabolic rate of glucose consumption. The small animal PET scanner used herein was the *easy*PET.3D system. This is a cost-effective benchtop PET system with a simple and unique image acquisition method (Patent, Universidade de Aveiro: WO2016147130), based on the rotation of two detector modules with two degrees of freedom (https://www.ri-te.pt/). This innovative scanning method, in which the detector modules are always face to face, strongly reduces parallax errors, thus simultaneously achieving a great level of detail and spatial resolution. Detector arrays can have different geometries. Each scan can be performed using different parameters to achieve different sensitivity, level of desired detail/speed, or image-specific regions of interest within the field of view (FOV), which is also a unique feature of this technology. The *easy*PET.3D model used in this study has two arrays of ^162^lutetium–yttrium oxyorthosilicate (LYSO) crystals with a size of 2,230 mm^3^ coupled to corresponding arrays of silicon photomultipliers with a 1.3-mm^2^ active area, covering an axial FOV of 3.4 cm (length) and a maximum radial FOV of 4.8 cm (diameter).

According to the experimental design (Figure 1), awake mice were i.p. injected with ^18^F-FDG (7.5 MBq/0.4 ml 0.9% NaCl) and placed in their home cages, after the behavioral test. For optimal radiotracer distribution, mice were kept conscious during the uptake period (60 min). Fifty minutes post-radiotracer injection, the animals were anesthetized (i.p.) [0.2 ml of a mixture of ketamine (1.5 mg/mg weight) + chlorpromazine (0.05 mg/mg weight) (3:1):saline (1:1)]. The anesthetized animals were placed on the bed of the *easy*PET.3D scanner, centered in the FOV. The PET imaging acquisition started, taking place during 30 min. A heating apparatus (Heat Therapy Pump, Adroit Medical Systems, Loudon, TN 37774, USA) is connected to the scanner's bed to keep the animals warm.

The data were reconstructed using a dedicated 3D reconstruction method based on a GPU implementation of the List-Mode Maximum-Likelihood Expectation-Maximization (LM-MLEM) algorithm, considering the original geometry of the *easy*PET.3D scanner and a high number of possible lines of response. The values of the PET image resulting from the reconstruction are expressed as a linear colormap (percent, %). Since cerebral metabolic rates of glucose consumption are reflected by local radiotracer uptake, a qualitative analysis of changes in brain metabolic activity of animals *non-exposed* or *exposed* to a single low dose of cocaine was done. In order to improve the image visualization, the hot metal scale was selected, and a threshold was applied. The Digimouse 3D mouse atlas (http://neuroimage.usc.edu) was applied for anatomical detail. Moreover, the volumes of interest (VOIs) were drawn from the same template co-registered with the PET data using AMIDE software (http://amide.sourceforge.net/). Semi-quantitative measurements of glucose metabolism were obtained using the standardized uptake value (SUV), which is a normalized target-to-background measure. SUV is defined as the regional tissue activity concentration (kBq/ml) normalized for both the decay correction of the injected activity (kBq) and weight of the studied animal (g). Usually, a density equivalent to 1.0 g/ml in tissue is assumed, ensuring that the units effectively cancel and the resulting SUV number becomes dimensionless. In the present study, the mean SUV was obtained, with the respective mean standard error correlated with the VOI. Areas too small to be identified using a microPET system were not included in this analysis.

### Histological Study

Macroscopy and microscopy analyses were done for the whole brain and different brain areas. The analyzed specimens were fixed in 10% neutral buffered formalin solution and processed for routine paraffin embedding. Three 4-μm sections were obtained from each block and stained with hematoxylin–eosin technique (H&E) for optical microscopy. The PFC pyramidal neurons, as well as the *hippocampus* (HC) and *cerebellum* (Cb) granular neurons, were counted by computerized image processing (NIS for Windows) (Martinez et al., 2011; Priolli et al., 2013). The number of neuronal cells was obtained as an average of three randomly selected fields of three sections from each animal.

### Statistical Analysis

The analysis of the results was performed by adopting a *p* < 5% (*p* < 0.05) to reject the null hypothesis, using the following statistical tools: sample size; descriptive statistics; measures of central tendency; normality test; comparison test (*t*-test); and two-way repeated-measures ANOVA followed by Sidak's multiple comparison tests (OFM study). The statistical Package Bioestat version 5.0 for Windows (Brazilian Science and Technology Ministry) was used.

## Results

### Behavioral Analysis

**Figure 2:**
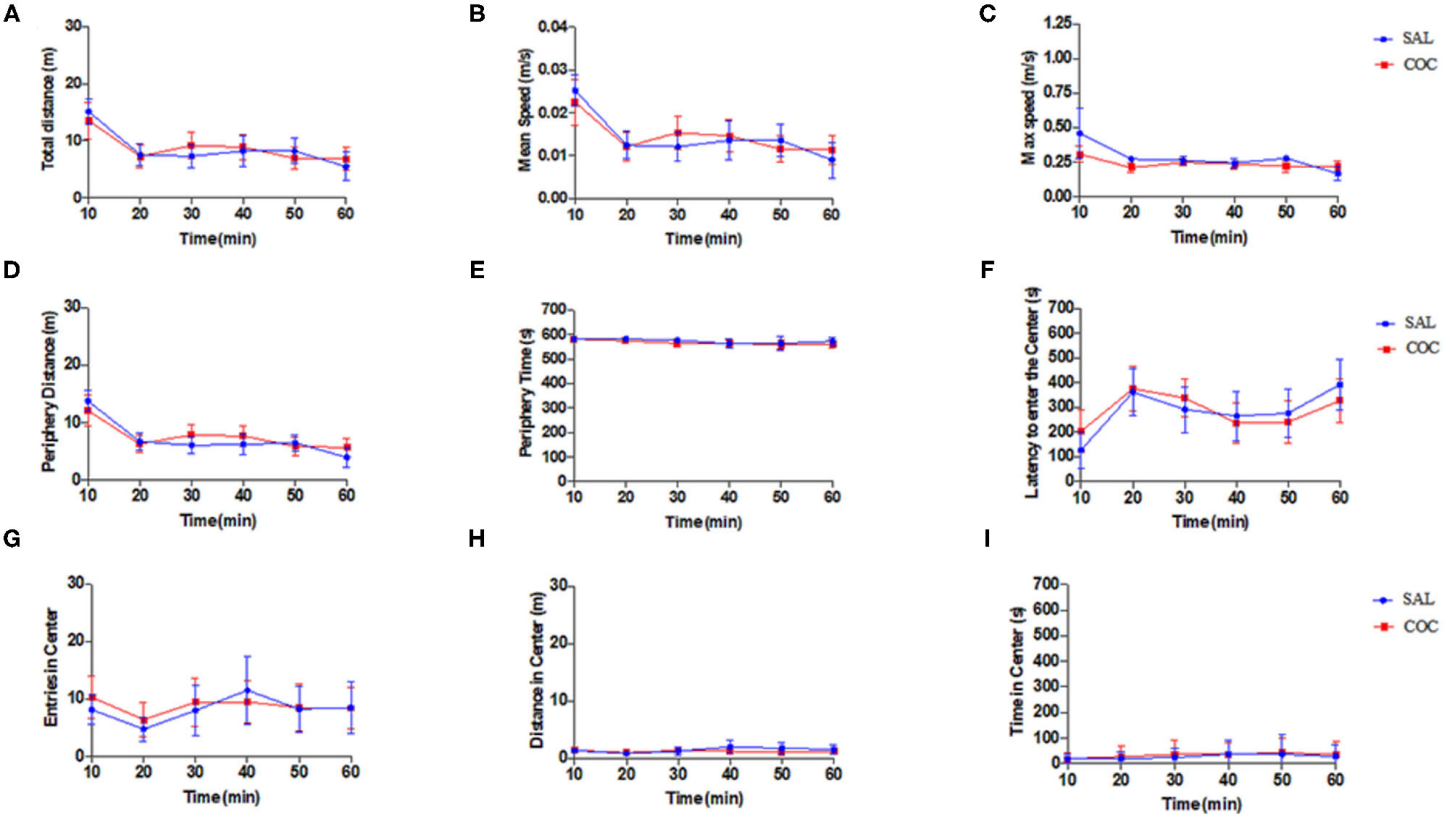
Open field behavior study. Mice *non-exposed* (saline injection, *n* = 8) and mice *exposed* (cocaine injection, *n* = 10) had similar behavioral parameters (two-way repeated-measures ANOVA followed by Sidak's multiple comparison tests): **(A)** total distance (m); **(B)** mean speed (m/s); **(C)** maximum speed (m/s); **(D)** periphery distance (m); **(E)** periphery time (s); **(F)** latency to enter the center (s); **(G)** entries in center; **(H)** distance in center (m); and **(I)** time in center (s). Data represent mean ± standard error of the mean.

Exploratory and locomotor activities of mice injected with a low dose of cocaine in an open field apparatus were evaluated. The OFM analysis showed no differences in the behavior of neither group of mice (*non-exposed* or *exposed* to cocaine). All analyzed parameters (including the total, peripheral, and central distance traveled; mean and maximum speed; time spent in the periphery and in the center; and time latency to enter in the center) were not statistically different between groups (*p* > 0.05) (Figure 2).

### Positron Emission Tomography Imaging Analysis

**Figure 3:**
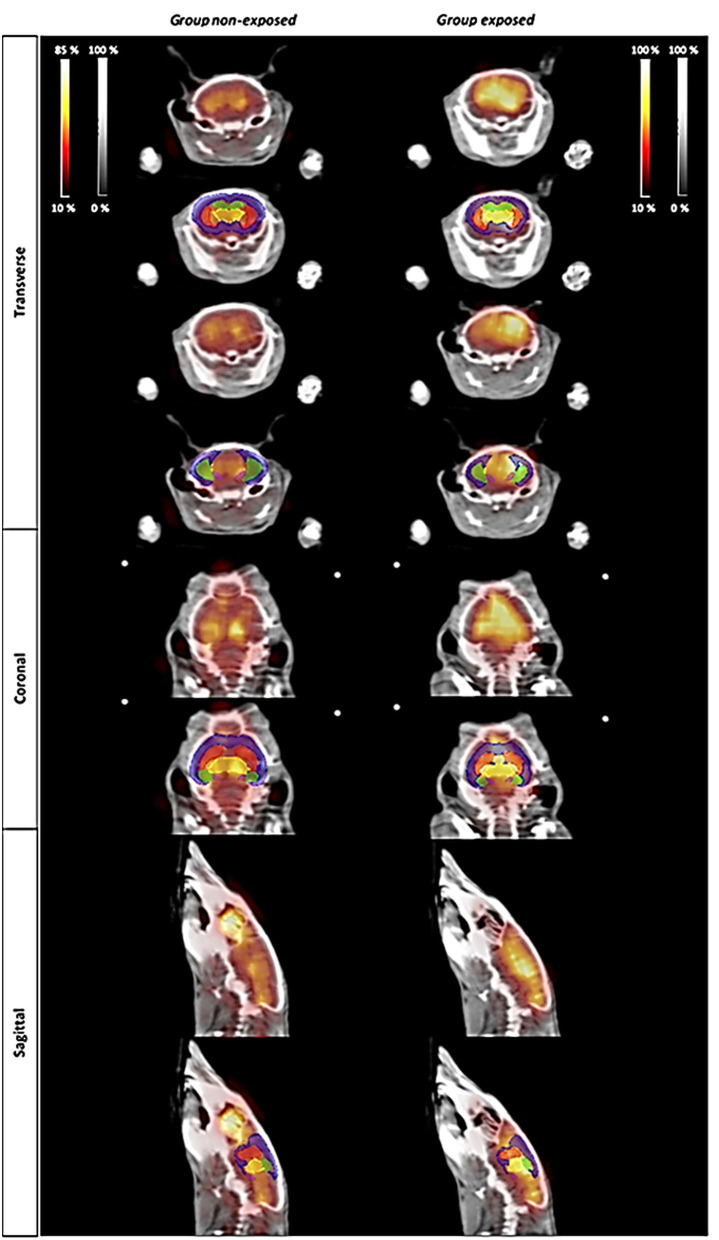
PET imaging study. Changes in metabolic activity in animals *non-exposed* (controls) and *exposed* to a single dose of cocaine (0.5 mg/kg). The atlas-derived volumes of interest (VOIs) of the main areas commonly affected by cocaine are shown superimposed on transverse, coronal, and sagittal image slices of mice brain from both representative ^18^F-FDG PET studies and CT derived from Digimouse 3D atlas. VOIs: prefrontal cortex (PFC, blue), *striatum* (St, red), *hippocampus* (HC, green), *thalamus* (TH, yellow), and *amygdala* (AMY, pink).

**Figure 4:**
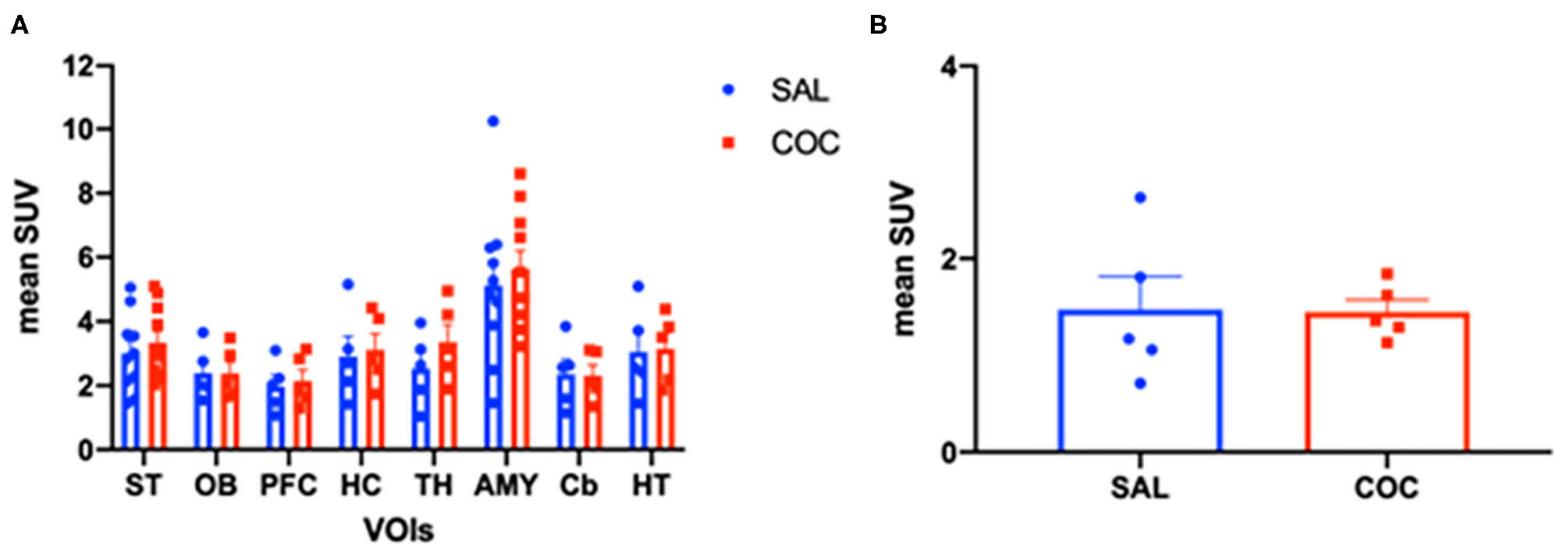
Bar graph of the statistical analysis of standardized uptake value (SUV) in animals *non-exposed* and *exposed* to a single low dose of cocaine. **(A)** SUV analysis of the following brain regions: *striatum* (ST), olfactory bulb (OB), prefrontal cortex (PFC), *hippocampus* (HC), *amygdala* (AMY), *cerebellum* (Cb), and *hypothalamus* (HT). **(B)** Metabolic all-brain analysis. Note the absence of metabolic changes. Data represent mean ± standard error of the mean.

Figure 3 illustrates the metabolic activity in mice *non-exposed* (saline injection) compared with mice *exposed* to cocaine. Representative ^18^F-FDG PET images were selected for each group (Figure 3). According to the intensity of the colormap selected (hot metal), the presence or absence of abnormal radiotracer accumulation was analyzed. The size and intensity of the uptake region, especially when the accumulation was focal, was also associated. The evaluation of the PET data (SUV) showed no significant differences between the groups (*non-exposed vs. exposed*) for any of the analyzed brain structures, which are typically affected by cocaine (Figure 4). Additionally, PET analysis of the entire brain showed no statistically significant alterations between groups (Figure 4).

### Histological Analysis

**Figure 5:**
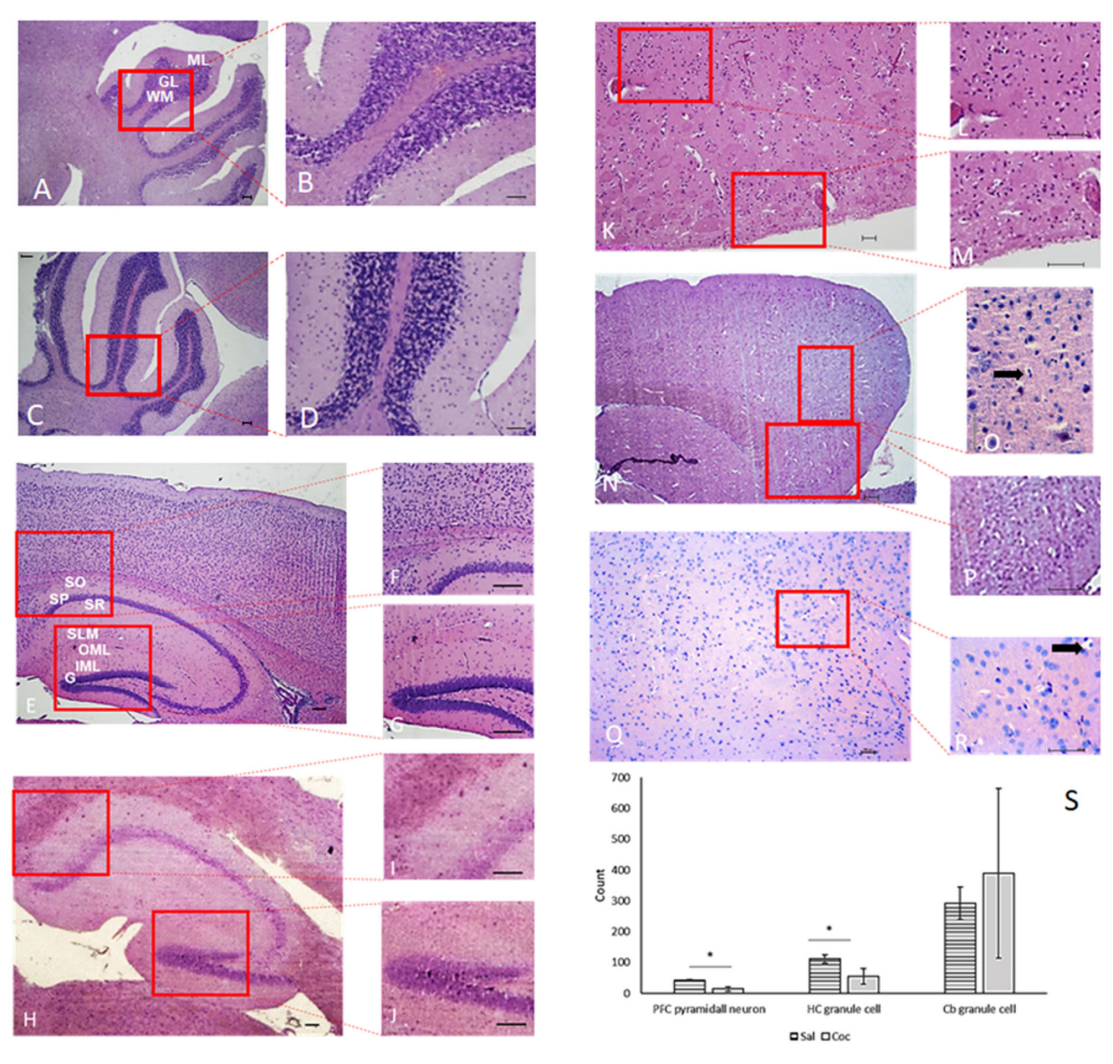
Microphotographs of PFC, HC and Cb. Cb **(A–D)** of saline **(A,B)**, and cocaine **(C,D)** animals. ML, molecular layer; G, granular layers; WM, white matter. Cocaine did not trigger any histopathological changes. HC **(E–J)** of saline **(E–G)**, and cocaine **(H–J)** groups. CA1 region **(F,I)** dentate gyrus **(G,J)**. SO, oriens layer; SP, pyramidal layer; SR, radiatum layer; SLM, lacunosum-moleculare layer; OML, outer molecular layer; IML, inner molecular layer; G, granule cell layer. Animals *exposed* to cocaine show hippocampal histopathological changes in the pyramidal cell layer and granule cell layer in the CA1 region and *dentate gyrus*, respectively, with neuronal loss **(G,H)**. PFC **(K–R)** of saline **(K–M)** and cocaine **(N–R)** groups. Animals depthwise to cocaine present histological changes, including ischemic necrosis **(P,Q)**. Observe (arrow) the clear halo around oligodendrocyte nuclei **(O,R)**. These features demonstrate irreversible hypoxic lesions and the presence of granule-adipose cells **(O,P)**. HandE: **(A**,**C**,**E**,**H**,**K**,**N**,**Q)**, 40×; **(F,G,I,J,L,M,P,R)**, 100×; **(B,D,O)**, 400×. **(S)** The difference between cell numbers in PFC (*p* = 0.008) and HC (*t*-test, n = 6, *p* = 0.05). Data represent mean ± standard deviation of the mean. PFC, prefrontal cortex; HC, hippocampus; Cb, cerebellum. *Significant.

Histological analysis of PFC, HC, and Cb were also performed in mice *non-exposed* and *exposed* to cocaine (Figure 5). No histological differences between groups were found for the Cb. On the contrary, morphological lesions were found in the PFC of mice *exposed* to cocaine, ranging from mild gliosis up to ischemic tissue necrosis. Additionally, histological analysis showed morphological deterioration and low neural count in PFC and HC in all animals exposed to cocaine. It is also noteworthy that the HC granular layer of the group *exposed* to a single low dose of cocaine was clearly distinct from that of controls.

## Discussion

Acute exposure to cocaine in humans includes euphoria, high self-confidence, motor arousal, restlessness, increased sensory perceptions, mood changes, irritability, impulsivity, anxiety, fear, paranoia, and avoidance (Silva et al., 2008). These symptoms are dependent upon the extension of cocaine impact to affected brain areas (Gallucci Neto et al., 2005). Although there is a robust body of literature regarding acute high doses of cocaine, as well as cocaine addiction scenarios, an integrated analysis of behavioral, metabolic, and structural brain changes associated with an acute low dose of cocaine is lacking.

Herein, the locomotor and exploratory behaviors associated with a low dose of cocaine using an OFM test were firstly analyzed. The presented behavioral data suggest that this cocaine dose (0.5 mg/kg) did not change either the locomotor or exploratory behaviors. It is well-known that this psychostimulant increase dose dependently the locomotor activity in different mice and rat strains (3–56 mg/kg) (Thomsen and Caine, 2011). For example, cocaine doses ranging from 1 to 20 mg/kg (Barr et al., 2020; Romero-Fernandez et al., 2020) have been shown to elicit psychomotor activating effects. da Silveira et al. (2018) also showed that 10 mg/kg of cocaine (but not lower doses including 2.5 and 5 mg/kg) increased the distance traveled by male Swiss mice in the open field (da Silveira et al., 2018). Moreover, it was further shown that the threshold dose of cocaine that significantly stimulated forward locomotion of rats in an open field arena was 10 mg/kg (Baumann et al., 2013). In the present study, 0.5 mg/kg was used (which is 20 times lower); therefore, it should not cause any locomotor or exploratory effect (the behavioral parameters evaluated in the open field arena). Thus, the absence of locomotor behavioral changes seen herein was expected. There is less information regarding the behavioral effects of cocaine in Balb-c mice, which is the strain used herein. It has been shown that 20 and 40 mg/kg of cocaine acutely induced locomotor activity in an open field arena for this mouse strain (Kosten et al., 2014; Murthy et al., 2014). However, the emotional and cognitive behaviors associated with cocaine for this mouse strain remain to be characterized. This should be done in future investigations.

The ^18^F-FDG PET imaging study performed herein showed no significant differences between controls and mice *exposed* to a single low dose of cocaine. This could be explained by the very low cocaine dose used. The experimental design could also be responsible for the absence of alteration in the PET analysis. In fact, PET-FDG images were acquired 1 h 50 min post-cocaine injection. It is noteworthy that it has been demonstrated that after i.p. injection of either 10 or 25 mg/kg of cocaine to mice, cocaine disappeared from the plasma and brain with a half-life of 16 min (Benuck et al., 1987). Therefore, the lack of metabolic changes that are seen here may reflect cocaine pharmacokinetics. In fact, this PET analysis may have been performed at a time point where there were only vestigial plasmatic cocaine levels. There are only a few studies looking at acute pharmacological effects of cocaine on rodent brain glucose metabolism. An acute intravenous administration of cocaine (0.75 mg/kg) decreased metabolic glucose rates in discrete brain areas (cortical and basal ganglia regions) of C57Bl/6 and DBA/2 awake mice (Zocchi et al., 2001). Nonetheless, the distribution pattern of these changes is different between the two strains. Briefly, in the referred study, rodents were sequentially intravenously injected with cocaine and with 2-[^14^C]deoxyglucose. Animals were sacrificed 40 min after the administration of the tracer, and brains were collected for glucose consumption assessment. These results are aligned with the findings in primates, also obtained using the quantitative 2-[^14^C]deoxyglucose method (Lyons et al., 1996). In fact, intravenous infusion of 1 mg/kg of cocaine acutely decreased glucose consumption in discrete brain structures including the interconnected limbic regions, such as ventral prefrontal cortex and ventral striatal complex in awake *Cynomolgus* monkeys. More recently, a PET-[^18^F]-FDG approach showed that cocaine (1 mg/kg) acutely increased metabolism in the prefrontal cortex, but not in the striatum of *Rhesus* monkeys in the cocaine-naïve state (Henry et al., 2010). These authors co-injected cocaine and [^18^F]-FDG and performed a static PET scan starting 40 min post-injection (image acquisition during 30 min). These apparently discrepant results may, however, highlight that cocaine acutely recruits cortical and subcortical regions and changes their metabolism in different species. Nonetheless, other studies are needed to see whether these metabolic alterations are long-lasting (e.g., 24, 48, 72 h, or 1 week later). Additionally, cocaine-induced activation was shown to be far less robust following withdrawal in a cocaine self-administration setting (Henry et al., 2010). This suggests that a history of cocaine use may impact the acute metabolic effects of cocaine. Finally, this absence of metabolic changes seems consistent with the lack of locomotor behavioral changes. In this context, the authors are already planning to perform PET scan analysis immediately after cocaine i.p. injection to examine its immediate pharmacological effects on glucose consumption.

Notably, cocaine induced histological alterations in PFC and HC, which are suggestive of mild gliosis up to ischemic tissue necrosis (Figure 5). Both PFC and HC have a crucial role in drug addiction processes, throughout the regulation of limbic reward regions and their involvement in higher-order executive and cognitive functions (e.g., self-control, salience attribution, and awareness; Goldstein and Volkow, 2011). The histological changes seen in this study raise concerns regarding episodic consumption of low doses of cocaine. Glutamate is the main excitatory neurotransmitter both in PFC and HC. A growing body of evidence suggests that cocaine indirectly influences glutamate transmission (Schmidt and Pierce, 2010). Therefore, one cannot rule out the role of glutamate in the cocaine-induced histological alterations reported here. Regarding the *hippocampus*, CA1 region is structured depthwise in defined layers: *oriens, pyramidale, radiatum*, and *lacunosum-moleculare*. The cell bodies of horizontal trilaminar cells and inhibitory basket cells are located in the *oriens*. *Pyramidale* layer contains the cell bodies of the pyramidal neurons, which are the main excitatory neurons of the *hippocampus*. This layer also contains the cell bodies of many interneurons, including axo-axonic cells, bistratified cells, and radial trilaminar cells. *Radiatum* layer contains commissural and septal fibers and Schaffer collateral fibers, which are projected to CA1. *Laconosum* is a thin layer and is often grouped with molecular stratum into a single layer named *lacunosum-moleculare layer*. Moreover, it contains Schaffer collateral fibers and perforant path fibers coming from the superficial layers of the entorhinal cortex. *Dentate gyrus* is part of the HC trisynaptic circuit and is thought to contribute to the formation of new episodic memories. This region promotes spontaneous exploration of novel environments, synaptic plasticity, rapid acquisition of spatial memory, and other functions (Saab et al., 2009; Lee et al., 2016). The CA1 and DG are the most sensitive regions to hypoxia, and their examination is mandatory to investigate possible acute neuronal necrosis and gliosis (Liu et al., 2004).

In fact, glial alterations were visible in the present study. The nuclei of glial cells are also recognizable in HE: the nuclei of astrocytes and oligodendrocytes are round, with the first being larger and more loose. The nuclei of the microglia are elongated, comma-shaped, and dense. When there is damage to the nervous tissue, the microglial cells lose their extensions and assume a rounded shape, constituting macrophages with phagocytic capacity. Histological analysis suggests that microglia in the cocaine group have fine foamy cytoplasm since they phagocytize lipids derived from degenerated nervous tissue. In *exposed* cocaine mice, histology showed gemistocytic astrocytes, characterized by abundant and pink cytoplasm and eccentric nuclei. A clear halo around oligodendrocyte nuclei can also be observed (Figure 5), suggesting the entry of water into the cells due to hypoxia.

Although there are few experimental studies about the Cb relationship to addictive drug behavior, evidence suggests that cerebellar activation may be involved in functions such as cognition, prediction, learning, and memory, being associated with compulsive and perseverative behaviors (Carbo-Gas et al., 2014; López-Pedrajas et al., 2015; Moreno-Rius and Miquel, 2017). The alterations in Cb resulting from chronic cocaine use have been correlated with its relationship and the maintenance of drug memory. However, despite the evidence of higher cerebellar activation in studies with cocaine, this mechanism is still unclear (Jiménez-Rivera et al., 2000; Nicastri, 2001; Carbo-Gas et al., 2014; López-Pedrajas et al., 2015; Vazquez-Sanroman et al., 2015). Cb neurons and glia are arranged in layers. The molecular layer is located at the surface and contains the dendrites of Purkinje neurons, axons of granule cells (parallel fibers), fibers of Bergmann glia, basket cells, and stellate cells. The granular cell layer contains granule cells, Golgi cells, Lugaro cells, and unipolar brush cells (Hashimoto and Hibi, 2012). In general, Cb has a characteristic dopaminergic distribution. Dopaminergic fibers, projecting from the ventral tegmental area to the cerebellar cortex, terminate mainly in the granular layer and additionally in the Purkinje cell layer, but not at all in the molecular layer (Ikai et al., 1992). This morphological characteristic can explain the absence of evident histological changes in animals exposed to cocaine. It may be consistent with findings suggesting a relationship between high doses of cocaine and gray matter volume reduction in the Cb (López-Pedrajas et al., 2015). One should stress that the histological alterations did not translate into locomotor and metabolic changes. This suggests that structural changes should be more profound and more widely spread across the brain to trigger functional brain changes. Nevertheless, the animal model presents some limitations, such as the inability to evaluate sociocultural and genetic factors, and personality and psychological traits, which are relevant issues to determine drug addiction development in humans (El Rawas et al., 2020). Future studies need to assess if these structural changes persist (e.g., 24, 48, 72 h, or 1 week later).

Overall, it is shown, for the first time, that a single low dose of cocaine, which did not change locomotor behavior and brain metabolism, has the potential to induce structural neurological damage. There is no safe dose for cocaine exposure. Brain structural changes must be considered regardless of the used dosage. It is essential to alert the population against any consumption, not underestimating acute and recreational dosage since the use, even in a low single dose, can generate structural tissue damage.

## Data Availability Statement

The raw data supporting the conclusions of this article will be made available by the authors, without undue reservation.

## Ethics Statement

The animal study was reviewed and approved by the ORBEA (Órgãos Responsáveis pelo Bem-Estar dos Animais (#17/2015) and the DGAV (DIREÇÃO DE SERVIÇOS DE PROTEÇÃO ANIMAL) authorization.

## Author Contributions

CN and DP: conception of the presented idea. CN: experiment execution (histological analysis, OFM, and PET). MP: experiment execution (OFM and PET). ACS: experiment execution (histological analysis and PET). FR and MP: experiment execution (PET). JR: experiment execution (OFM). ML: experiment execution (histological analysis). CN, DP, and FR: wrote the manuscript. CN, FR, FP, ACS, and DP: interpretation of the results. FP and MB: helped supervise the project. JV, ACS, and DP: supervision of the project. All authors discussed the results and contributed to the final manuscript.

## Conflict of Interest

The authors declare that the research was conducted in the absence of any commercial or financial relationships that could be construed as a potential conflict of interest.
